# La Grippe

**Published:** 1890-02

**Authors:** S. W. Cohen

**Affiliations:** Waco, Texas


### LA GRIPPE.

S. W. Cohen, M. D., Waco, Texas.

I may not be doing justice to the large number of your readers when I utilize your valuable pages for the purpose of descanting upon such an inconsequential condition (from a homœopathic standpoint) as la grippe, but when I receive reports which show that even in so-called homœopathic medical associations, Quinine has been suggested and indorsed for this strange epidemic with a French name, and when I reflect that I know many would be, if could be, or rather could be, if would be, homœopathists who use Quinine in material doses in almost every case, I think it no more than proper that a Hahnemannian report should find its way into print, to shame those who are sailing under false colors, not only to their own detriment and that of those who are confided to their medical care, but to the prejudice of Homœopathy, as understood and taught by the master.

The grippe first made its bow to our community about two weeks since, though we had a forerunner of the trouble in the guise of a peculiar vertigo. Possibly six weeks ago my wife complained of intense dizziness from the time she arose in the morning until she retired at night. This continued for a week, and no medicine that I prescribed seemed to bring relief from the unpleasant sensation. The following week I was attacked in a similar manner, and I permitted the annoyance to wear itself away. With this vertigo there came a confused and oppressed condition of the brain—I could not study or think. The remedies apparently indicated failed to bring any amelioration. Many of my friends were subject to the same symptoms, but none thought them of sufficient moment to ask medical advice.

This vertiginous attack was certainly the *avant courier* of "la grippe," and was, no doubt, induced by similar atmospheric influence. My wife was among the first to succumb to the fashionable intruder. Her symptoms began to develop an hour or two after noon, and were as follows: Incessant sneezing, a nasal discharge of clear, hot fluid, which soon inflamed the alæ nasi and spread to the cheeks. There was a raw, burning sensation from trachea down to the end of the sternum. She received a single dose of Arsenicum$^{mm}$ (F.) at about five or six o'clock P. M., and was perfectly well next morning. I did not take her temperature. A few evenings following I retired in my usual good health, but was awakened—I know not at what time during the night—by severe chills running up and down my back, and down my thighs. No amount of cover relieved.

I dropped asleep and was again awakened by a chill, accompanied by a sharp pain running from my left foot to my thigh. There were several recurrences of this attack before daylight. During the following day I suffered with headache, rheumatic (?) pains in lower limbs, wrists, finger-joints and lumbar region, the most intense pain being in the neighborhood of the Gasserian ganglion. I bore it like a martyr until evening, when I was forced to lie down, but no position suited me. I hovered about the fire, stretching my aching limbs. Wife begged me to take some medicine. I was restless, pains worse on first moving, but walking about the room awhile relieved, until I was again forced to rest. Ameliorated by covering up warmly: wanted even my head covered, and desired to lie with my back to the fire. The weather was cold and damp. Asked wife to get me a dose of Rhus-tox., and she brought me Rhus$^{dmm}$ (Swan). I was easier in fifteen minutes, and fell asleep. Was entirely recovered by next day, though even now I am troubled with vertigo daily. My temperature was normal throughout. I cite these two cases as samples of many others. The major portion of cases under my care had no catarrhal symptoms. Many adult patients had fever, but others none. Children attacked, invariably had fever, and in all cases treated by me, with but one exception, in which the temperature rose to 104°, the thermometer indicated 102° or 102½°. *Every one of these cases* was treated with Bell.$^{cm}$ (F.), the larger number receiving but a single dose of the remedy, and recovering in from twelve to twenty hours. In no case was a second visit necessary. The only adult to whom Bell. was given was the mother of the babe whose temperature rose to 104°. She was screaming with headache, and was asleep within five minutes from the time she received a dose of the CM of Bell., and up and about bright and happy the following morning. In no case have I observed any complications or sequelæ—no bronchitis, no pneumonia, and but transient debility. The drugs employed during the epidemic are Acon.$^{cc}$ (Dunham), Bell.$^{cm}$ (F.), Eup.-perf.$^{cc}$, Baptisia$^{45m}$ (F.), Colch.$^{m}$, Rhus-tox.$^{dmm}$ (Swan), and Arsenicum$^{mm}$ (F.), according to indications.

So much for treatment under the guidance of that infallible law, *Similia similibus curantur*, influenced by that other, and corollary legend, *Simplex simile minimum*.

